# A Global Perspective of Color Vision Deficiency: Awareness, Diagnosis, and Lived Experiences

**DOI:** 10.3390/healthcare13162031

**Published:** 2025-08-17

**Authors:** Ali Almustanyir

**Affiliations:** Optometry Department, College of Applied Medical Sciences, King Saud University, Riyadh 11362, Saudi Arabia; aalmustanyir@ksu.edu.sa

**Keywords:** color vision deficiency, awareness, color vision diagnosis, red–green vision

## Abstract

Color vision deficiency (CVD), commonly referred to as color blindness, affects a significant portion of the global population, particularly among males. This narrative review synthesizes findings from peer-reviewed articles and published large-scale epidemiological studies identified through database searches using terms such as “color vision deficiency,” “color blindness,” “awareness,” and “diagnosis.” Studies were included if they addressed prevalence, awareness, diagnosis, or lived experiences of individuals with CVD. The prevalence of CVD varies by population, with red–green CVD affecting up to 8% of males and 0.5% of females of Northern European descent and lower rates reported in Asian and African populations. Although CVD is congenital in most cases, diagnosis is often delayed until school age or later due to limited routine screening, with many individuals remaining unaware of their condition until adolescence or adulthood. This delay can result in significant educational, occupational, and psychosocial challenges. This review synthesizes the current literature on the prevalence of CVD, levels of awareness, the age and process of diagnosis, and the lived experiences of individuals affected by this condition. Recommendations are provided for early detection, educational adaptations, and societal support.

## 1. Introduction

Color vision is a complex and vital aspect of human sensory perception, fundamentally shaping how individuals interact with their environment, interpret visual cues, and engage in daily activities [1,2]. The ability to distinguish between different wavelengths of light enables humans to perceive a rich spectrum of colors. This capability is essential not only for aesthetic appreciation but also for practical tasks such as reading maps, interpreting traffic signals, and recognizing warning signs [3]. However, a significant proportion of the global population experiences color vision deficiency (CVD), a condition characterized by an impaired ability to differentiate certain colors most commonly along the red–green axis [4,5].

CVD is predominantly an inherited disorder, with most cases arising from X-linked recessive genetic mutations that impact the photopigments in retinal cone cells [4,5]. Consequently, the prevalence of CVD is substantially higher in males, affecting up to 8% of men of Northern European descent, compared with less than 1% of women [5]. While red–green deficiencies (protan and deutan types) constitute the vast majority of cases, blue–yellow (tritan) deficiencies and total color blindness (achromatopsia) are relatively rare [4,5].

Despite its high prevalence, CVD is often underdiagnosed and poorly understood by the public or by healthcare professionals [6,7]. Many individuals remain unaware of their condition until later in life, frequently discovering it through incidental screenings or challenges with color-dependent tasks [8,9,10,11,12]. This lack of awareness can adversely affect education and career opportunities, particularly in professions requiring accurate color discrimination [13,14,15].

Recent advances in clinical testing, digital screening tools, and public health initiatives have improved the detection and management of CVD [5,16,17,18,19,20,21,22,23,24,25,26]. Nevertheless, significant gaps remain in early identification, public awareness, and the provision of appropriate educational and occupational accommodations [27,28]. Understanding the lived experiences of individuals with CVD, along with the factors that influence their awareness and adaptation, is crucial for developing effective support strategies and promoting social inclusion. Existing reviews often focus on clinical aspects or prevalence data but rarely synthesize evidence on the real-world impact of delayed diagnosis, the effectiveness of current screening practices, or the adequacy of educational and occupational accommodations. Furthermore, there is limited exploration of how cultural, regional, and systemic factors influence awareness and support for individuals with CVD. This review aims to address these gaps by providing a comprehensive synthesis of current epidemiological data, examining barriers to timely diagnosis and support, and highlighting the personal and societal challenges faced by those with CVD. By integrating clinical, educational, and psychosocial perspectives, this work seeks to inform more effective strategies for early detection, inclusive education, and workplace adaptation, ultimately promoting equity and social inclusion for individuals with color vision deficiency. Special emphasis is placed on the educational and occupational implications of CVD, the psychosocial impact of delayed or missed diagnoses, and evidence-based recommendations for screening and support.

In addition to its clinical and social challenges, the relatively stable prevalence of color vision deficiency especially the approximately 8% frequency among males of Northern European descent suggests potential evolutionary significance. Several hypotheses propose that certain forms of CVD may confer selective advantages in ancestral environments. For instance, individuals with color vision deficiencies might have been better able to detect camouflaged objects or subtle texture contrasts in foliage, aiding in foraging or hunting activities [1,2]. Such advantages could help maintain CVD-associated genetic variants at moderate frequencies through balancing selection or heterozygote advantage. Including these evolutionary perspectives enriches our understanding of why CVD persists despite its disadvantages in modern settings.

## 2. Epidemiology and Types of CVD

### 2.1. Prevalence

CVD is one of the most common inherited visual disorders worldwide, with prevalence rates that vary significantly across different populations, sexes, and ethnicities [4,5]. Most cases are congenital and result from mutations in the genes that encode the photopigments of retinal cone cells, primarily affecting the red–green color axis [4,5].

#### 2.1.1. Global Prevalence and Sex Differences

Large-scale epidemiological studies consistently indicate that CVD is significantly more prevalent in males than in females. In populations of Northern European ancestry, the prevalence of red–green CVD is estimated at approximately 8% in males and 0.5% in females [5,29,30,31,32,33,34,35,36]. This sex disparity is observed globally, although the absolute rates may vary by region and ethnic background. Table 1 presents data from different resources showing the estimated prevalence percentages of CVD [5,15,17,37].

The lower prevalence of certain X-linked genetic disorders in females compared with males is due to the inheritance pattern of genes located on the X chromosome. Females possess two X chromosomes (XX), while males have one X and one Y chromosome (XY). For a female to express an X-linked recessive disorder, she must inherit two copies of the defective gene—one from each parent. In contrast, males need only to inherit a single mutated gene on their sole X chromosome to be affected, as they do not have a second X chromosome with a potentially normal copy of the gene to compensate [38,39,40].

This genetic mechanism indicates that males are significantly more likely to express X-linked recessive disorders, such as hemophilia A, Duchenne muscular dystrophy, and red–green CVD [4,5,38,39,40]. Females with only one mutated gene are typically carriers; they usually do not exhibit symptoms because the normal gene on their other X chromosome can compensate for the defective one. However, in rare cases where a female inherits two mutated copies (one from each parent), she will also express the disorder [4,5,38,39].

#### 2.1.2. Ethnic and Geographic Variation

The prevalence of CVD is not uniform across all populations. Birch highlights that red–green CVD is most common among individuals of European descent, while lower rates are reported in Asian and African populations [5]. For example, studies conducted in Nigeria and India report lower prevalence rates compared with those typically observed in Western countries [37]. In a study of schoolchildren in the Republic of Ireland, the prevalence among males was 4.7%, with no cases detected among females [41]. Similarly, a study in Durban, South Africa, found a prevalence of 2.2% among boys and none among girls [42].

The reported prevalence of CVD varies markedly across studies, largely due to both genetic factors and differences in study design, diagnostic methods, and population demographics. Most studies use standardized color vision screening, such as the Ishihara pseudoisochromatic plates, but the choice of test, criteria for diagnosis, and age groups included can vary. Some studies focus on school-aged children, while others assess occupational groups or community samples, resulting in differences in age, sex ratio, and ethnic background. These methodological and population-related differences must be taken into account when comparing published prevalence rates (Table 1). Therefore, direct comparison between studies should be done cautiously, as variability in screening method, diagnostic criteria, and study population can affect the estimates.

#### 2.1.3. Acquired Color Vision Deficiency

While most cases of CVD are congenital, acquired CVD can arise as a secondary condition due to ocular or systemic diseases, medication toxicity, or environmental exposures [4,24,40]. However, acquired forms are less common and do not significantly contribute to overall prevalence statistics.

#### 2.1.4. Causes

Acquired CVD can result from a wide range of factors, including [4,5,7,11,14].

- Ocular Diseases: Conditions such as glaucoma, age-related macular degeneration, cataracts, diabetic retinopathy, and retinitis pigmentosa can impair color vision by affecting the retina or optic nerve.
- Neurological Diseases: Disorders that affect the brain or optic pathways, such as multiple sclerosis, Alzheimer’s disease, Parkinson’s disease, and strokes, can lead to color vision loss.
- Medications and Toxins: Certain drugs (e.g., hydroxychloroquine, ethambutol, antibiotics, and barbiturates) and exposure to industrial or environmental chemicals (e.g., carbon monoxide, carbon disulfide, and lead) have been implicated in causing acquired CVD.
- Trauma: Injuries to the eye or brain, including retinal detachment or tumors, can disrupt normal color perception.
- Aging: Natural aging processes, particularly after the age of 60, can lead to a gradual decline in color vision, which often accelerates after the age of 70.

#### 2.1.5. Clinical and Public Health Implications

The high prevalence of CVD, particularly among males, has significant clinical and public health consequences that extend across educational, occupational, and safety domains.

1. Educational Implications

CVD can profoundly affect educational experiences, especially during early childhood and adolescence when color-based learning materials are commonly used. Students with undiagnosed CVD may face challenges in interpreting color-coded information, reading maps, or participating in laboratory experiments, potentially resulting in academic underachievement or misdiagnosis of learning difficulties. Therefore, early identification of CVD is essential, as it enables educators to provide appropriate classroom accommodations, such as the use of patterns, labels, or high-contrast materials, and to tailor instructional strategies accordingly. Furthermore, increasing awareness among teachers and school counselors can facilitate informed career guidance, helping students avoid professions where normal color vision is a critical requirement [4,16,40].

2. Occupational Health Considerations

CVD has notable implications for occupational health and workforce planning. Many professions, including those in transportation, electrical work, healthcare, and graphic design, require accurate color discrimination for safety and quality assurance. Individuals with undiagnosed CVD may be inadvertently placed in roles where their condition could compromise both their own safety and that of others. Routine vision screenings during occupational health assessments can help identify affected individuals, enabling employers to implement reasonable accommodations, such as alternative labeling systems or assistive technologies. Additionally, career counseling for individuals with CVD can guide them toward suitable professions, thereby reducing the risk of occupational hazards and enhancing job satisfaction.

3. Public Safety Concerns

CVD poses several public safety challenges, particularly in sectors where accurate color perception is critical, such as transportation, emergency services, and industrial work environments.

Individuals with CVD may experience difficulty distinguishing traffic signals, brake lights, and warning signs, which can affect their ability to respond promptly to road hazards. For instance, studies have shown that color-deficient drivers, especially those with protanopia, may have a reduced capacity to detect red stoplights or taillights, particularly in low-light conditions or while wearing sunglasses. This impairment can result in delayed reaction times and potentially increase the risk of rear-end collisions, especially on slippery roads where stopping distances are longer [15,43]. Additionally, CVD drivers often report challenges in distinguishing between traffic signals and may confuse traffic lights with streetlights [15,43].

Despite these challenges, large-scale studies and regulatory reviews indicate that CVD does not significantly increase the overall risk of traffic accidents. Adaptive strategies such as memorizing the standardized positions of traffic lights and relying on cues other than color enable many color-deficient drivers to compensate for their limitations. Consequently, accident rates among CVD drivers are not significantly higher than those of individuals with normal color vision [14,15]. However, awareness of their condition and education about compensatory strategies are crucial for maintaining safety [14,15].

In occupational settings, especially in industries that rely on color-coded safety signals (e.g., factories, electrical work, and laboratories), CVD can increase the risk of errors that may endanger both the individual and others. For example, misinterpreting colored warning lights or electrical wires can lead to accidents or hazardous situations. Therefore, routine color vision screening and workplace accommodations such as shape-coded signals or alternative labeling are recommended to mitigate these risks [44,45,46].

4. Underdiagnosis and Awareness

Despite its frequency, CVD remains underdiagnosed in many settings, largely due to the absence of routine screening and limited awareness among educators, healthcare providers, and the general public [16,38,40]. This underdiagnosis can lead to missed opportunities for early intervention and support. Public health initiatives should prioritize increasing awareness of CVD and implementing systematic screening programs in schools, workplaces, and healthcare settings. Enhanced recognition and documentation of CVD can inform policy development and resource allocation, ultimately improving outcomes for affected individuals. Addressing the needs of those with CVD is not just a matter of individual accommodation; it requires a societal commitment to inclusion. Proactive identification and support for individuals with CVD foster a more inclusive society, enabling all individuals to reach their full potential regardless of their color vision status [14,15,47,48].

### 2.2. Types of Color Vision Deficiency

CVD is classified according to the specific photopigment defect and the affected cone type in the retina. The main categories include red–green deficiencies, blue–yellow deficiencies, and total color blindness, each exhibiting distinct genetic and clinical characteristics [4,5,42]. Table 2 summarizes the CVD types.

#### 2.2.1. Red–Green Color Vision Deficiency

Red–green CVD is the most prevalent form, accounting for approximately 95% of all congenital cases [4,5]. It is inherited in an X-linked recessive manner, which explains its significantly higher prevalence in males [5]. There are two principal subtypes:

- Protan Deficiency: This condition arises from anomalies or the absence of long-wavelength (L) cones, which are most sensitive to red light. Individuals with protanomaly (reduced sensitivity) or protanopia (complete absence) experience difficulty distinguishing between red and green hues, and reds may appear darker than normal [4,42]. Protan defects are less common than deutan defects, accounting for about 1% of males in European populations [5].
- Deutan Deficiency: Deutan defects result from anomalies or the absence of medium-wavelength (M) cones, which are most sensitive to green light. Deuteranomaly (reduced sensitivity) and deuteranopia (complete absence) similarly impair red–green discrimination, but they do not cause the darkening of reds observed in protan defects [4,42]. Deutan anomalies represent the most common form of CVD, affecting up to 6% of males in certain populations [5]. Both protan and deutan deficiencies can vary in severity, ranging from anomalous trichromacy (partial color discrimination) to dichromacy (complete loss of one type of cone) [4].

#### 2.2.2. Blue–Yellow Color Vision Deficiency (Tritan Deficiency)

Blue–yellow CVD, or tritan deficiency, is considerably rarer and affects both sexes equally, as it is inherited in an autosomal dominant pattern [4,42]. Tritan defects arise from anomalies in the short-wavelength (S) cones, which are responsible for blue light perception. Individuals with tritanomaly (reduced sensitivity) or tritanopia (complete absence) experience difficulty distinguishing between blue and green, as well as between yellow and violet hues [4]. The prevalence of tritan defects is estimated to be less than 0.01% of the population [5].

#### 2.2.3. Total Color Blindness (Achromatopsia)

Achromatopsia is a rare and severe congenital CVD characterized by the complete absence of functional cone photoreceptors. This condition results in monochromatic vision, where individuals perceive the world only in shades of gray. In addition to total color blindness, affected individuals commonly experience photophobia (light sensitivity), nystagmus (involuntary eye movements), and markedly reduced visual acuity, often ranging from 20/100 to 20/200 [4,42]. The condition is inherited in an autosomal recessive manner, affecting both males and females equally [4,49,50,51].

Diagnosis is typically confirmed through clinical examination and electroretinography, which reveals absent or severely diminished cone responses while preserving rod function. Optical coherence tomography may show structural retinal abnormalities, such as foveal hypoplasia. Although achromatopsia is generally non-progressive, the visual impairment significantly impacts daily functioning. Current management is supportive, focusing on symptom relief through tinted lenses and low-vision aids. Advances in gene therapy offer promise for future treatments aimed at restoring cone function [50,51].

#### 2.2.4. Blue-Cone Monochromacy

Blue-cone monochromacy (BCM) is a rare congenital disorder characterized by the complete absence of functional long-wavelength (L) and medium-wavelength (M) cones. Individuals with BCM retain only functioning short-wavelength (S, “blue”) cones and rods. Clinically, BCM results in severely impaired color discrimination—limited to shades distinguishable by S-cone function—accompanied by reduced visual acuity, photophobia, and nystagmus. Unlike achromatopsia, in which all cones are affected, individuals with BCM have some residual color vision along the blue–yellow axis. BCM is inherited in an X-linked recessive pattern and predominantly affects males.

## 3. Awareness and Age of Diagnosis

### 3.1. General Awareness

Awareness of CVD remains generally low among affected individuals, particularly during childhood. Many children are unaware of their condition until they face specific challenges in academic or occupational settings, such as difficulties with color-coded materials or tasks that require color discrimination [6]. This lack of awareness is consistently observed across different populations.

For example, a study conducted in Nigeria revealed that only 19.2% of students with CVD were aware of their condition, while the vast majority (80.8%) were not [52]. Similarly, research from Karachi, Pakistan, reported a significant lack of awareness among children in primary school diagnosed with CVD, emphasizing the need for routine screening upon school entry to facilitate early identification and support. Additionally, an investigation in India found that although 75.2% of surveyed students had heard of CVD, only a small minority could accurately define it. Furthermore, knowledge of CVD was strongly linked to the adoption of practical precautions, such as regular eye checkups and the use of the Ishihara chart [53].

These findings are echoed in other regions as well. In a study from Ethiopia, the majority of children with CVD were unaware of their color vision status, reinforcing the recommendation for continuous visual screening programs in schools [30].

The low awareness of CVD among schoolchildren underscores the importance of implementing regular color vision screenings and educational initiatives in schools. Early identification enables timely accommodations and guidance, helping affected children adapt and make informed decisions about their education and future careers [30,53,54].

A critical comparison of international approaches reveals that health systems and educational policies significantly influence awareness, diagnosis, and outcomes for individuals with CVD. In countries with established school-based vision screening programs, such as the United Kingdom, Japan, and parts of Scandinavia, CVD is often detected early in childhood, enabling timely educational accommodations and informed career guidance. Early identification allows teachers to adapt materials and support students, reducing academic disadvantage and psychosocial stress. In contrast, in regions where routine screening is not mandated or is inconsistently implemented, such as many low- and middle-income countries, CVD frequently goes undiagnosed until adolescence or adulthood, often only after individuals encounter repeated difficulties with color-dependent tasks or occupational barriers. This delayed recognition can lead to prolonged frustration, lower self-esteem, and restricted career opportunities. Furthermore, public awareness campaigns and educator training are more prevalent in countries with formal screening policies, contributing to a more supportive environment for individuals with CVD. These disparities underscore the importance of integrating systematic screening and targeted awareness initiatives within both health and educational systems to promote early detection, equitable access to support, and improved long-term outcomes for those affected by CVD.

### 3.2. Age and Diagnostic Process

**Figure 1:**
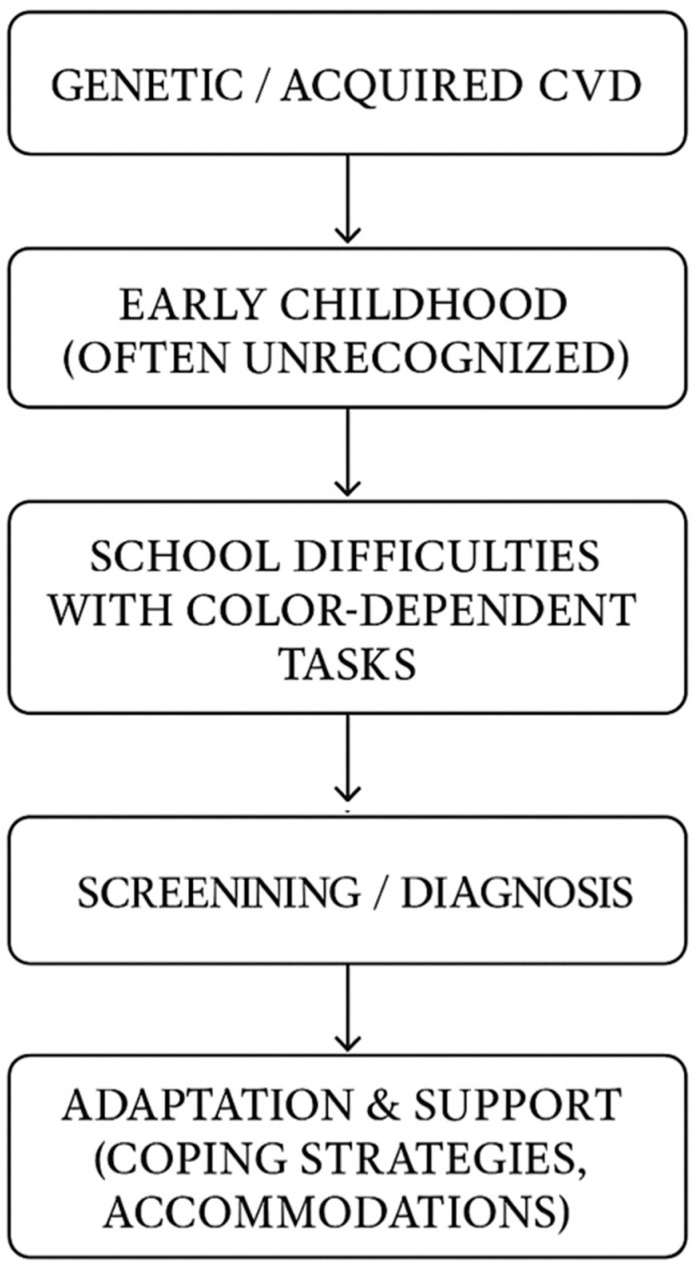
Pathway to color vision deficiency (CVD) diagnosis.

CVD is frequently not detected until late childhood or adolescence, often after children encounter challenges with color-dependent tasks in school or during routine vision screenings [16,40,41,55,56]. Many children with CVD remain unidentified in their early years because the signs can be subtle and easily overlooked by parents and teachers. Early indicators may include unusual or inconsistent color choices in drawings, confusion when using color-coded materials, or difficulty following instructions that rely on color cues [57]. Figure 1 is a pathway representation to CVD diagnosis.

Children may be mistakenly perceived as inattentive or underperforming when, in reality, their difficulties stem from an inability to distinguish certain colors. As a result, they may be incorrectly grouped in lower academic tracks or experience frustration and stigma. The lack of early detection is compounded by the fact that routine color vision screening is not universally implemented in schools, and many parents remain unaware of the condition until academic issues arise [55,56,57].

Testing for CVD can reliably begin between the ages of 4 and 6, as children in this age range are typically able to participate in standardized color vision tests [30,47,58]. Early identification is important, as it enables timely educational accommodations and helps prevent misunderstandings about a child’s abilities, thereby supporting both academic achievement and emotional well-being [30,47,58].

## 4. Educational and Occupational Impact

### 4.1. Educational Experiences

Children with undiagnosed CVD often experience repeated academic challenges, particularly in tasks involving color-coded materials, as well as in science and art subjects [10,42]. These difficulties can lead to frustration, reduced self-esteem, and negative educational outcomes [6,12]. Table 3 illustrates the impact of CVD on various aspects of academic performance.

Several studies have highlighted the educational impact of undiagnosed CVD. Children with CVD may struggle with classroom activities that require color discrimination, such as interpreting charts, maps, or laboratory materials. This struggle can undermine their confidence and hinder their learning [36,47]. The lack of early detection is often attributed to the absence of routine color vision screening in many school health programs, as well as limited awareness among teachers and parents regarding the signs of CVD [36].

Reliable color vision testing is feasible for children aged 4–6 years, and implementing such screening as part of school health initiatives is recommended to facilitate early identification and support [36]. Early diagnosis enables timely educational accommodations and counseling, helping affected children adapt and make informed decisions about their educational paths and future careers [36].

### 4.2. Occupational Limitations

CVD imposes significant occupational limitations, especially in professions where accurate color discrimination is critical for safety and effective job performance. Fields such as aviation, electrical work, transportation, the armed forces, and certain areas of healthcare and engineering frequently require normal color vision as a condition of employment [60,61]. For example, pilots must accurately interpret color-coded signals and navigational aids, electricians must distinguish between colored wires, and transportation workers must rely on color-coded systems to ensure safety and operational efficiency [60,61].

CVD may restrict access to certain professions, particularly those that require accurate color discrimination, such as pilots, electricians, and transportation workers [13,14]. Regulatory standards for color vision vary by country and occupation [15,62,63]. Table 4 summarizes the professions commonly restricted for individuals with CVD.

The impact of CVD on career opportunities is well documented. A study from Australia found that 34% of individuals with CVD reported that their career choices were influenced by their color vision status, and 24% were explicitly barred from specific occupations, such as police, military, railways, and electronics, due to their deficiency [60]. In India, individuals with CVD may only be offered temporary positions in certain medical specialties, reflecting the stringent vision standards required in fields like cardiology, pathology, and radiology [60].

Regulatory standards for color vision vary considerably across countries and occupations. For instance, the UK Civil Aviation Authority has introduced the Color Assessment and Diagnosis (CAD) test, which allows a broader range of individuals with CVD to qualify as pilots compared with previous standards. In contrast, some countries maintain strict exclusion policies, while others have adopted more pragmatic, competency-based assessments to determine fitness for specific roles [60,65]. In Canada and the US, there is no universal legal requirement for normal color vision in most occupations; however, individual employers or sectors may impose their own standards [7,61].

These occupational restrictions underscore the importance of early detection and counseling for individuals with CVD. Such measures empower them to make informed career choices and, where possible, seek accommodations or alternative pathways within their chosen fields [7,60,61,65].

## 5. Psychosocial Experiences and Adaptation

### 5.1. Coping Strategies

Many individuals with CVD develop compensatory strategies, such as memorizing the order of traffic lights, using contextual clues, or seeking assistance from others [11,14,41]. Technological aids, such as color identification apps, are increasingly being employed [36]. Table 5 shows the common coping strategies utilized by individuals with CVD.

### 5.2. Psychosocial Impact

Unrecognized CVD can lead to social misunderstandings, embarrassment, and stigmatization, particularly in group or classroom settings [12,67]. Repeated errors may be misattributed to carelessness or a lack of effort, which can negatively affect self-esteem and peer relationships [6,11].

Individuals with CVD often report a range of psychosocial challenges stemming from their condition. For example, many discover their deficiency incidentally sometimes after repeated difficulties in school or at work—leading to feelings of frustration or embarrassment when they are unable to perform tasks that peers complete with ease [6,11]. One qualitative study participant described, “I always felt anxious during art class because I could never be sure if I was using the right colors. It made me want to avoid those activities altogether” [65]. Such experiences can contribute to diminished self-esteem, particularly when color-based mistakes are misinterpreted by teachers or colleagues as carelessness or lack of effort [65].

Despite these efforts, the lack of public awareness and formal accommodations can lead to social exclusion or limited career opportunities. For instance, one interviewee shared, “I wanted to be a pilot, but when I failed the color vision test, I felt like a door had closed before I even had a chance to try” [65]. These experiences highlight the importance of early diagnosis, open communication, and inclusive practices in both educational and occupational environments.

Incorporating such real-world perspectives, direct quotes, and descriptions of coping and support mechanisms will provide a richer, more empathetic understanding of the lived experience of CVD and strengthen the psychosocial dimension of a research review [68,69,70].

Children with CVD may feel embarrassed or isolated, which can lead to a decline in self-esteem and confidence. The stigma associated with making “avoidable” mistakes can foster feelings of frustration, isolation, and anxiety, especially if the child is unaware of the underlying cause of their difficulties. Peer relationships may also be adversely affected, as classmates may tease or exclude children who consistently struggle with color-based tasks or games. Over time, these negative experiences can contribute to a reluctance to participate in classroom activities or group projects, further impacting academic performance and social integration.

The psychosocial impact of CVD extends beyond childhood. Adults with undiagnosed or unacknowledged CVD may continue to encounter misunderstandings in both workplace and social environments, which can affect career progression and strain interpersonal relationships. Therefore, raising awareness and ensuring early diagnosis is crucial not only for implementing practical accommodations but also for supporting the emotional well-being and self-acceptance of individuals living with CVD.

## 6. Screening, Diagnosis, and Educational Support

Routine color vision screening, especially during early childhood, is recommended to facilitate timely diagnosis and support [8,9]. The Ishihara test remains the most widely used screening tool; however, newer digital and tablet-based tests are emerging [5,16,17].

**Figure 2:**
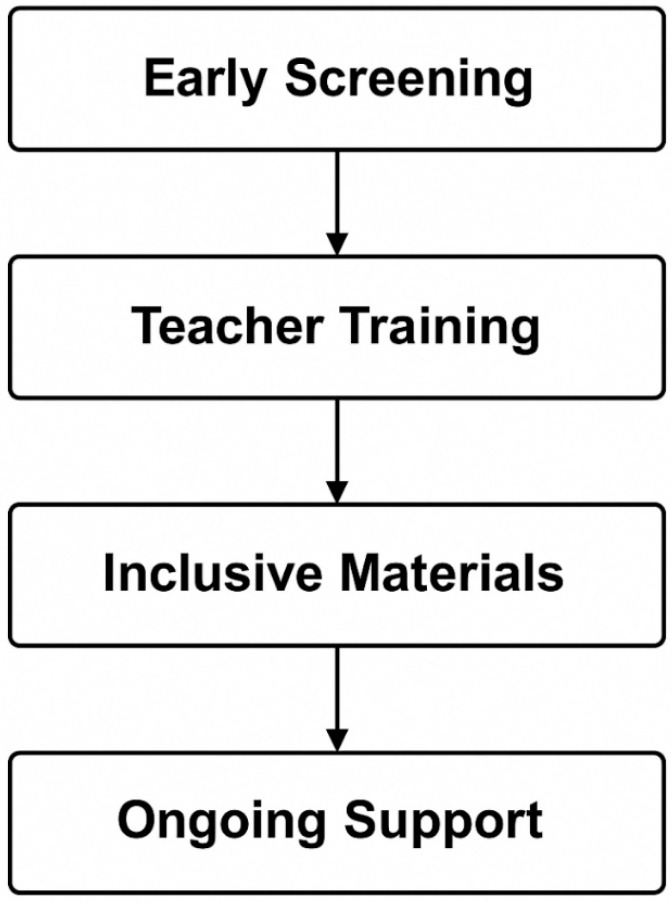
Framework practices for supporting students with color vision deficiency (CVD).

Figure 2 shows suggested framework practices for supporting students with CVD. The best practices for assisting students with CVD include using high-contrast colors and patterns—not color alone—to convey information [27], providing teacher training to recognize and accommodate CVD [27], and offering alternative materials and exam accommodations [10]. Table 6 presents practical recommendations for educators, clinicians, and employers based on study findings and guidance.

## 7. Limitations

This review has several limitations that should be acknowledged. First, as a narrative synthesis, the selection of studies may be subject to publication bias, with a tendency for positive or novel findings to be overrepresented in the literature. Second, there is a lack of longitudinal studies examining the long-term educational, occupational, and psychosocial outcomes for individuals with CVD, which limits the ability to draw conclusions about changes over time. Third, much of the available research is concentrated in high-income countries, resulting in underrepresentation of data from low- and middle-income regions, where awareness, screening practices, and support systems may differ significantly. Additionally, studies focusing on the lived experiences of individuals with CVD are relatively scarce, and qualitative data are limited. These factors may affect the generalizability of the findings and highlight the need for further research across diverse populations and settings.

## 8. Future Directions

Advances in genetic research and assistive technology are poised to significantly enhance the management of CVD [71,72]. Ongoing genetic studies are deepening the understanding of the underlying causes of CVD, paving the way for earlier diagnosis and personalized interventions and potentially for gene-targeted therapies. Meanwhile, rapid progress in assistive technology, including wearable devices, adaptive software, and augmented reality tools, offers new avenues for supporting independence and daily functioning among individuals with CVD.

Despite these promising scientific and technological developments, societal awareness and inclusive practices remain critical. Raising public understanding of CVD can help dismantle misconceptions, reduce stigma, and foster environments that accommodate diverse visual needs. Schools, workplaces, and public spaces must continue to adopt inclusive policies and accessible designs to ensure that individuals with CVD can fully participate in all aspects of life.

Looking ahead, a multidisciplinary approach that integrates medical advancements, technological innovations, and societal inclusion will be essential. Collaboration among researchers, clinicians, educators, policymakers, and advocacy groups will facilitate the translation of scientific progress into meaningful improvements in the quality of life for those affected by CVD.

## 9. Conclusions

CVD is a common, lifelong condition that has significant implications for education, employment, and psychosocial well-being. Awareness of this condition remains suboptimal, and many individuals are diagnosed only after experiencing functional difficulties. Early screening, increased public and professional awareness, and inclusive educational and occupational practices are essential to minimize the impact of CVD and support affected individuals.

## Figures and Tables

**Table 1:** Prevalence of CVD by sex and region.

Population/Region	Males (%)	Females (%)
European Caucasians	8	0.4
Chinese	4.0–6.9	<1
Japanese	~4.0	<1
Druze Arabs	10	NA
Aboriginal Australians	1.9	NA
Fijians	0.8	NA
DR Congolese	1.7	NA
Indians (Andhra Pradesh)	7.5	NA
Norwegians	9	NA
Russians	9.2	NA
Northern Europe/USA	8.0	0.5
Nigeria (Imo State)	4.7	1.1
India (Hyderabad)	1.33	0.25
Republic of Ireland	4.7	NA
South Africa (Durban)	2.2	NA

Data adapted from [5,15,17,37]. Reported prevalence rates are influenced by the diagnostic criteria, screening methods, and demographic characteristics of the studied populations.

**Table 2:** Summary of types of color vision deficiency.

Type	Affected Cones	Main Color Confusions	Frequency	Prevalence	Inheritance
Protanopia	L (red) absent	Red–black, red–green	Most common (M)	~1% males	X-linked recessive
Deuteranopia	M (green) absent	Green–red, green–brown	Most common (M)	~6% males	X-linked recessive
Protanomaly	L (red) weak	Red appears dull/greenish	Common	<0.01% population	Autosomal dominant
Deuteranomaly	M (green) weak	Green appears redder	Most common	Very rare	Autosomal recessive
Tritanopia	S (blue) absent	Blue–green, yellow–pink	Rare	Variable	N/A
Tritanomaly	S (blue) weak	Blue–green, yellow–red	Very rare		
Achromatopsia	All cones	Total color loss	Very rare		Autosomal recessive
Blue-cone monochromacy	L and M absent; S only	Severe; only blue hues and grayscale	Very rare		X-linked recessive
Acquired	Any	Any axis	Variable	Variable	N/A

**Table 3:** Impact of CVD on academic performance.

Task Type	Reported Difficulty (CVD)	Reference
Color-coded assignments	High	[42]
Science/Art subjects	High	[59]
Reading colored graphs	Moderate to High	[10]

**Table 4:** Professions commonly restricted for individuals with CVD.

Profession	Color Vision Requirement
Aviation (pilots)	Strict [13]
Railway/Transport	Strict [63]
Electrical work	Moderate to strict [15]
Healthcare	Variable [64]

**Table 5:** Common coping strategies among individuals with CVD.

Strategy	Prevalence (%)
Memorizing color positions	High [11]
Asking others for help	Moderate [12]
Using technology/apps	Increasing [66]

**Table 6:** Practical summary table of recommendations for educators, clinicians, and employers based on findings and guidance presented in this study.

Stakeholder	Practical Recommendations
Educators	- Implement routine color vision screening in early school years - Use patterns, textures, or labels instead of color alone for instructional materials and assessments - Avoid relying solely on color-coded information in teaching aids, maps, and charts - Provide alternative assignments or support for color-dependent tasks - Raise awareness among teachers and school counselors to guide students with CVD toward suitable career paths
Clinicians	- Include color vision assessment in standard pediatric and adult eye exams - Educate patients and families about the implications of CVD - Document CVD status in medical records for future reference - Offer guidance on coping strategies and available assistive technologies - Refer to genetic counseling when appropriate
Employers	- Conduct color vision screening for roles where color discrimination is safety-critical - Provide reasonable workplace accommodations (e.g., alternative labeling systems, shape-coded signals, assistive technology) - Ensure safety protocols account for employees with CVD - Offer career counseling and guidance regarding job suitability - Foster an inclusive work environment by raising awareness about CVD and reducing stigma

## Data Availability

Not applicable.
